# Professional Quality of Life Among Institution-Based Psychologists in Ecuador: A Multilevel Cross-Sectional Study of Compassion Satisfaction, Burnout, and Secondary Traumatic Stress

**DOI:** 10.3390/bs16091669

**Published:** 2026-09-17

**Authors:** Marina R. Ramírez, Mercy P. Ontaneda

**Affiliations:** Department of Psychology, Universidad Técnica Particular de Loja, Loja 110107, Ecuador; mpontaneda@utpl.edu.ec

**Keywords:** professional quality of life, mental health professionals, psychological distress, occupational wellbeing, institutional clustering, Ecuador

## Abstract

Evidence on professional quality of life among psychologists in Latin America remains limited. This multicenter cross-sectional study examined compassion satisfaction, burnout, and secondary traumatic stress among 1078 Ecuadorian psychologists nested within 92 institutions using nonprobability sampling. Participants completed the Professional Quality of Life Scale, Version IV (ProQOL-IV), and the 12-item General Health Questionnaire (GHQ-12). Random-intercept linear mixed-effects models were estimated. Unconditional ICCs were 0.086, 0.097, and 0.139, respectively. Greater psychological distress was associated with lower compassion satisfaction (B = −0.605, 95% CI [−0.674, −0.535]) and higher burnout (B = 0.407, 95% CI [0.354, 0.461]) and secondary traumatic stress (B = 0.906, 95% CI [0.843, 0.969]); all *p* < 0.001. No secondary sociodemographic or occupational association survived the Benjamini–Hochberg correction (minimum q = 0.163). Findings support cautious, non-diagnostic monitoring and longitudinal research directly assessing institutional job demands and resources. Cross-sectional design and nonprobability sampling limit causal inference and generalizability.

## 1. Introduction

### 1.1. Occupational Context and Current Challenges for Psychologists

Psychologists routinely work with emotional suffering, trauma histories, chronic illness, disability, and other forms of clinical and psychosocial vulnerability. Sustained empathic engagement can make this work deeply meaningful, but repeated exposure to complex clinical situations may also accumulate as emotional strain and psychological distress (Ben-Zur & Michael, 2007; Figley, 1995, 2002; Kercher et al., 2024; Santana Ferreira & Zaia, 2025). The magnitude of this concern is not trivial: Kercher et al. (2024) summarized pre-pandemic estimates indicating that approximately 20% to 67% of psychologists experienced burnout symptoms. Indirect exposure to clients’ traumatic experiences is additionally relevant because it may be accompanied by intrusion, avoidance, arousal, fear, and other trauma-related reactions described as secondary traumatic stress (Bride, 2007; Pellegrini et al., 2022). These patterns make it important to consider both the occupational costs and the sustaining rewards of psychological helping work rather than treating professional well-being as a single favorable-unfavorable continuum.

### 1.2. Job Demands-Resources Framework and Professional Quality of Life

The Job Demands-Resources (JD-R) model provides a theoretical framework for understanding why demanding helping work can lead to different professional experiences. The model proposes a health-impairment process in which persistent job demands consume physical and psychological effort, alongside a motivational process in which job resources support engagement, coping, and sustained functioning (Bakker & Demerouti, 2007). For psychologists, emotionally intense clinical encounters, workload, and indirect exposure to trauma can operate as salient demands, whereas social support, autonomy, supervision, resilience, and other organizational or personal resources may help professionals manage those demands (Aminihajibashi et al., 2024; Singh et al., 2020; Ventouris et al., 2025). This framework is clinically relevant because prolonged imbalance between demands and available resources can affect both practitioner well-being and the sustainability of high-quality psychological care.

Professional quality of life captures both positive and negative experiences that can emerge from sustained helping work. The ProQOL model distinguishes compassion satisfaction, burnout, and secondary traumatic stress as related but non-equivalent dimensions (Deriglazov et al., 2025; Jaramillo-Cartwright et al., 2025; Lobo et al., 2024; Stamm, 2010). Compassion satisfaction reflects the sense of meaning, effectiveness, and reward derived from helping others (Maurya & DeDiego, 2025). Burnout reflects chronic exhaustion and strain associated with persistent occupational demands (Bakker, 2026), whereas secondary traumatic stress concerns trauma-related reactions arising from indirect exposure to the experiences of people receiving care (Bride, 2007; Yılmaz & Bekaroğlu, 2025). These dimensions can coexist rather than representing opposite ends of one continuum (Craig & Sprang, 2010; Niemitz et al., 2022; Rossi et al., 2012; Sprang et al., 2007). Their simultaneous relevance is illustrated by a large 2022 study of Ecuadorian health-care providers, in which 84.9% were classified in the high compassion-satisfaction range while 57.1% and 59.6% were classified in the moderate burnout and secondary-traumatic-stress ranges, respectively (Jaramillo-Cartwright et al., 2025). Although those pandemic-era findings involved the broader health-care workforce and should not be transferred directly to psychologists, they show that substantial professional strain can coexist with a strong sense of meaning and reward in Ecuadorian care settings.

### 1.3. Psychological Distress and Professional Quality of Life

General psychological distress is clinically relevant because it reflects broad disturbances in emotional well-being and everyday functioning that can include difficulties with mood, anxiety, sleep, concentration, confidence, and coping (Goldberg & Williams, 1988). It is broader than occupation-specific experiences such as burnout or secondary traumatic stress, making their relationship important for understanding whether unfavorable professional quality of life is accompanied by more generalized mental-health strain. Psychologist-specific research has documented elevated psychological distress, burnout, secondary traumatic stress, and risk factors for compassion fatigue under periods of sustained work pressure (Kercher & Gossage, 2024; Kercher et al., 2024). In Ecuador, a pandemic-era study of 1056 health-care workers reported that 66.0% met the study’s GHQ-12 criterion for psychological distress (Ruiz-Frutos et al., 2022). Although that estimate reflects an exceptional historical period and a broader health-care population, it underscores the local clinical importance of examining psychological distress together with professional quality of life.

### 1.4. Ecuadorian Context, Knowledge Gap, and Study Objectives

Evidence on professional quality of life among psychologists remains concentrated largely in high-income settings. In low- and middle-income countries, resource constraints, staffing conditions, and organizational environments may shape exposure to occupational strain, yet Ecuadorian research has focused mainly on physicians, nurses, and health-care workers more broadly rather than psychologists (Gómez-Salgado et al., 2021; Habtu et al., 2025; Ndulue et al., 2024; Phu et al., 2026; Rosales Vaca et al., 2022; Ruiz-Frutos et al., 2022; Vaca-Auz et al., 2024). Recent Ecuadorian evidence indicates that compassion satisfaction, burnout, secondary traumatic stress, and psychological distress are relevant concerns in the national health-care workforce (Jaramillo-Cartwright et al., 2025; Ruiz-Frutos et al., 2022), but psychologist-specific multicenter evidence remains limited. Pandemic-era studies also provide historical context for heightened emotional and organizational demands among mental-health professionals, while the present post-pandemic data are not interpreted as direct consequences of COVID-19 (Cuartero-Castañer et al., 2021; Delle Donne et al., 2023; Glowacz et al., 2022; Kercher et al., 2024).

The study therefore had three objectives: (1) to describe compassion satisfaction, burnout, and secondary traumatic stress among institution-based psychologists in Ecuador; (2) to estimate the proportion of variability in each ProQOL-IV dimension attributable to differences between institutions; and (3) to examine adjusted associations between psychological distress and each ProQOL-IV dimension while accounting for institutional clustering and prespecified sociodemographic and occupational covariates. No a priori directional hypotheses were specified.

The corresponding research questions were (a) how are the three ProQOL-IV dimensions distributed in the sample; (b) how much of their variability is attributable to institutions; and (c) how is psychological distress associated with each dimension after adjustment for institutional clustering and sociodemographic and occupational characteristics? Secondary analyses examined the same sociodemographic and occupational characteristics.

## 2. Materials and Methods

### 2.1. Study Design and Setting

This observational, cross-sectional, multicenter study examined institution-based psychologists working in public, private, and mixed-sector services across Ecuador. Data were collected between November 2024 and March 2025. Ninety-four institutions in the capitals of the country’s 24 provinces were contacted to obtain broad territorial and institutional coverage; 92 institutions were represented in the final analytical sample. The institutions included services operating at different levels of care, but the recruitment procedure was nonprobabilistic and was not intended to produce a statistically representative sample of all Ecuadorian institutions or psychologists.

Work setting was self-reported in the sociodemographic and occupational questionnaire. Participants were asked to define the area in which they worked and selected either urban or rural. This variable therefore reflects participants’ characterization of their primary work setting; the research team did not subsequently classify work setting using residence, institutional headquarters, population size, or an external geographic standard.

The manuscript was prepared in accordance with the Strengthening the Reporting of Observational Studies in Epidemiology (STROBE) recommendations for cross-sectional studies (von Elm et al., 2007). Institution-specific denominators for potentially eligible psychologists were not provided to the research team under participating institutions’ data-protection and confidentiality procedures; consequently, institution-specific participation or response rates could not be calculated.

### 2.2. Participants

Eligible participants were psychologists actively providing direct psychological care in institution-based services, with at least one year of professional experience, and with a current professional license. Their work included assessment, diagnosis, psychological intervention, mental-health promotion, and related clinical activities across primary, secondary, and tertiary levels of care. Individuals who were absent from their position during data collection or who did not provide informed consent were not eligible for the analytical sample.

Nonprobability sampling with planned coverage by province and institution type was used (Hulley et al., 2013). Public, private, and mixed-sector institutions were contacted through their authorities or administrators, and participation depended on institutional authorization and voluntary participation by eligible psychologists. Because there was no probability sampling frame or known selection probability, coverage of all 24 provinces should not be interpreted as national representativeness.

The initial sample-size calculation used Epidat version 4.2 (Dirección Xeral de Saúde Pública, Xunta de Galicia, Santiago de Compostela, Spain) for the descriptive objective of estimating the proportion of participants with high compassion satisfaction. Assuming an expected proportion of 10%, absolute precision of 2%, 95% confidence, and 20% anticipated loss yielded a minimum of 865 participants. The calculation was not designed to provide model-specific power for the subsequent mixed-effects analyses and did not incorporate a design effect for institutional clustering. This a priori calculation reflected the original descriptive recruitment-planning criterion; the three ProQOL-IV outcomes are reported and analyzed continuously in the final analyses.

### 2.3. Procedure

A standardized protocol specified recruitment, eligibility, measures, data management, quality-control procedures, and ethical safeguards. A preliminary pilot with 60 psychologists from six private healthcare institutions in Loja assessed feasibility and clarity; pilot participants were not included in the analytical sample. Research assistants received 20 h of training on eligibility, informed consent, standardized administration, and confidentiality.

**Figure 1:**
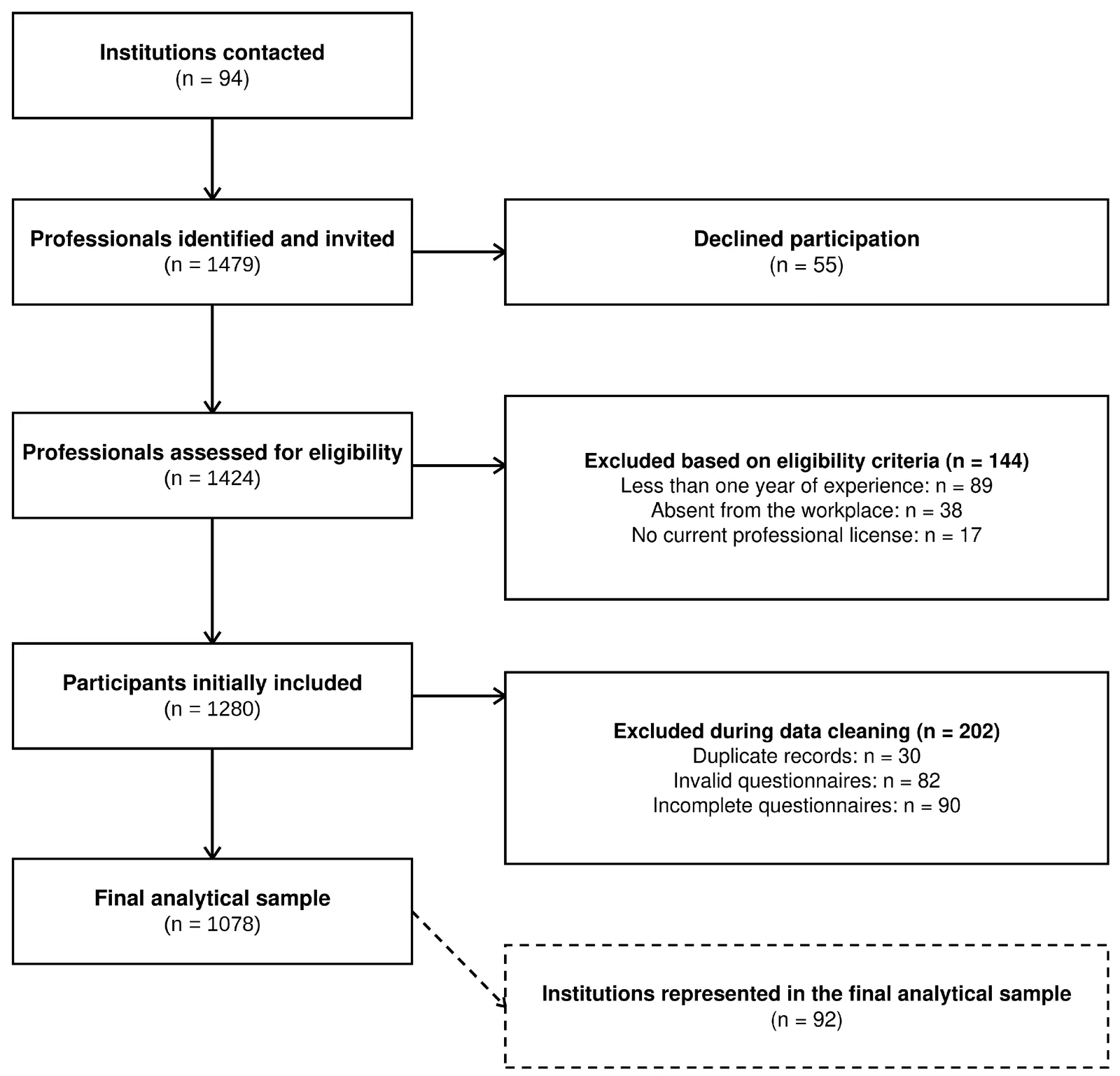
Flow of institutional contact, recruitment, eligibility assessment, and formation of the analytical sample. The n values indicate the number of institutions or professionals, as applicable. The final analytical sample comprised 1078 psychologists from 92 institutions. The dashed arrow and box indicate the institutional composition of the final analytical sample and do not represent an additional participant-selection or exclusion step.

Institutions were contacted formally, and psychologists who potentially met eligibility criteria were invited to participate voluntarily. Neither institutions nor participants were randomly selected. The assessment battery was administered online through the ArcGIS Survey123 web app (version 3.21; Esri, Redlands, CA, USA) after electronic informed consent. Mean completion time was approximately 20–25 min. Prespecified data-cleaning procedures addressed duplicate records, invalid or inconsistent questionnaires, and incomplete records; the numbers excluded at each stage are reported in Results and Figure 1.

### 2.4. Measures

#### 2.4.1. Sociodemographic and Occupational Characteristics

Participant characteristics were assessed using an ad hoc questionnaire covering sex, age, marital status, employment sector, contract type, employment status, monthly income, professional experience, shifts, daily working hours, and self-reported work setting. For work setting, participants were asked to define the area in which they worked and select urban or rural; no external geographic classification was applied.

#### 2.4.2. Professional Quality of Life Scale (ProQOL-IV)

Professional quality of life was assessed with the 30-item Professional Quality of Life Scale, Version IV (ProQOL-IV; Stamm, 2005), Spanish version by Morante-Benadero et al. (2006). The instrument yields three 10-item scores—compassion satisfaction, burnout, and secondary traumatic stress—each ranging from 0 to 50, with higher values indicating more of the corresponding dimension. Items are rated from 0 (never) to 5 (always), and the burnout score includes the reverse coding specified in the Version IV instructions. Subscale scores were analyzed continuously and were not interpreted as clinical or diagnostic categories. Complete scoring syntax is provided in Supplementary Material S1. In the present sample, reliability was alpha = 0.89 and omega = 0.90 for compassion satisfaction, alpha = 0.73 and omega = 0.77 for burnout, and alpha = 0.89 and omega = 0.89 for secondary traumatic stress. Previous confirmatory factor-analytic research supported a three-factor ProQOL structure in Spanish- and Portuguese-speaking palliative care professionals (Galiana et al., 2017). Complementary validation of a short form based on ProQOL-IV/V likewise supported a correlated three-factor solution and measurement invariance across three national samples (Galiana et al., 2020). These findings support analyzing the ProQOL dimensions separately, although they do not constitute validation specifically among Ecuadorian psychologists.

#### 2.4.3. General Health Questionnaire (GHQ-12)

General psychological distress was assessed with the 12-item General Health Questionnaire (GHQ-12; Goldberg & Williams, 1988). The Likert scoring method (0-1-2-3) was used, with positively worded items reverse-coded so that higher total scores (range 0–36) indicated greater psychological distress. The score was analyzed continuously and was not used to establish diagnoses or clinical categories.

The GHQ-12 has shown adequate psychometric properties in Ecuadorian university students (Moreta-Herrera et al., 2018). In the present sample, internal consistency was high (alpha = 0.915; omega = 0.92), and corrected item-total correlations ranged from 0.563 to 0.727. Exploratory internal-structure analyses supported the operational use of a global score, while not constituting confirmatory validation specifically among Ecuadorian psychologists.

### 2.5. Ethical Considerations

The study was conducted in accordance with the Declaration of Helsinki and was approved by the Human Research Ethics Committee of Universidad Técnica Particular de Loja, Ecuador (protocol code CEISH-UTPL-(09)-(2024)-No. 08). Informed consent was obtained from all participants, and the confidentiality of their responses was ensured. Participation was voluntary, and no financial or other compensation was provided.

### 2.6. Statistical Analysis

All analyses were conducted in IBM SPSS Statistics for Windows, Version 26.0 (IBM Corp., Armonk, NY, USA). The primary inferential analyses used random-intercept linear mixed-effects models estimated with restricted maximum likelihood (REML), a variance-components covariance structure, and the Satterthwaite approximation for degrees of freedom.

Descriptive analyses summarized continuous variables using means, standard deviations, and ranges and categorical variables using frequencies and percentages. ProQOL-IV dimensions and GHQ-12 psychological distress were analyzed as continuous scores. No imputation or score prorating was performed; descriptive, correlational, and multivariable analyses used the final complete-case sample of 1078 participants.

Because 1078 participants were nested within 92 institutions, unconditional linear mixed-effects models with a random intercept for institution were first estimated for compassion satisfaction, burnout, and secondary traumatic stress. Variance components were estimated using REML. The ICC was calculated as institutional variance/(institutional variance + residual variance) and is interpreted as a descriptive estimate of between-institution clustering; no formal comparison among ICC point estimates was performed.

Bivariate associations were examined using Pearson correlations for continuous variables, point-biserial correlations for continuous-dichotomous pairs, and phi coefficients for dichotomous pairs. Institution identifiers were not treated as quantitative variables. A focused correlation table for the main study variables is presented in the manuscript, and the complete correlation matrix remains in Supplementary Material S3.

For the adjusted analyses, three random-intercept linear mixed-effects models were estimated, one for each ProQOL-IV dimension. Fixed effects were sex, marital status, work setting, contract type, employment status, employment sector, monthly income, daily working hours, professional experience, and GHQ-12 score. Monthly income was rescaled per USD 100.

For the adjusted models, unstandardized coefficients, REML/Satterthwaite-based 95% confidence intervals, and two-sided *p*-values are reported. Institutional and residual variances and adjusted ICCs are also presented. The Benjamini–Hochberg procedure was applied as a multiplicity adjustment to the 27 secondary sociodemographic and occupational effects (nine predictors across three outcomes); GHQ-12 was the primary psychological predictor and was not included in this 27-test family. Secondary associations were interpreted as exploratory regardless of nominal *p*-values when they did not retain q < 0.05.

Age and professional experience were highly correlated (r = 0.834) and were not entered simultaneously; professional experience was retained because of its closer relation to cumulative clinical work. Correlations among the retained individual-level variables are provided in Supplementary Material S3.

Residual and influence diagnostics for the primary models are provided in Supplementary Material S2. These diagnostics evaluated linearity, homoscedasticity, residual distributions, extreme observations, and institutions with extreme random intercepts. Sensitivity analyses re-estimated the models after excluding extreme observations and, separately, institutions with extreme random intercepts.

### 2.7. Generative AI Use in Manuscript Preparation

During manuscript preparation and revision, ChatGPT (GPT-5.6 Sol; OpenAI) was used for English-language drafting and editing, restructuring text in response to peer-review comments, formatting, and editorial consistency checks. It was not used for study design, data collection, statistical analysis, or generation or alteration of study data. All AI-assisted text was reviewed and revised by the authors, who take full responsibility for the manuscript.

## 3. Results

### 3.1. Participant Flow, Sample, and Institutional Characteristics

Of 1479 psychologists identified as potentially eligible and invited, 55 declined participation, and 1424 were assessed for eligibility. A total of 144 were excluded at eligibility assessment (89 with less than one year of experience, 38 absent during data collection, and 17 without a current professional license), leaving 1280 initially included participants. During prespecified data cleaning, 202 questionnaires were removed (30 duplicates, 82 invalid or inconsistent records, and 90 incomplete questionnaires), resulting in a final complete-case analytical sample of 1078 psychologists from 92 institutions (Figure 1).

Sample characteristics are summarized in Table 1. Women comprised 55.5% of the sample, mean age was 36 years (SD = 9.3), 88.1% self-reported an urban work setting, and 84.6% worked full-time. Mean GHQ-12 score was 10.1 (SD = 6.8). Institution-specific response rates could not be calculated because institution-level denominators were not available to the research team.

### 3.2. Distribution of ProQOL Scores

Mean scores were 38.1 (SD = 8.8) for compassion satisfaction, 24.1 (SD = 6.6) for burnout, and 14.9 (SD = 9.6) for secondary traumatic stress. Observed score ranges are presented with the continuous summaries in Table 2; no clinical or diagnostic categories were assigned.

### 3.3. Institutional Clustering of Professional Quality of Life Dimensions

The 1078 participants were distributed across 92 institutions. Cluster size ranged from 10 to 16 participants per institution, with a median of 12 and an interquartile range of 1 (Q1 = 11; Q3 = 12). Cluster sizes were relatively homogeneous: all institutions had at least 10 participants, and 11 had exactly 10.

The unconditional models indicated between-institution variability in all three ProQOL-IV dimensions. Institutional variance/residual variance and ICCs were 6.756/71.459 and 0.086 for compassion satisfaction, 4.268/39.754 and 0.097 for burnout, and 12.729/79.024 and 0.139 for secondary traumatic stress (Table 3). The ICC point estimate was numerically highest for secondary traumatic stress; however, uncertainty intervals for the variance components were not estimated, so these point estimates were not formally compared.

### 3.4. Bivariate Correlations

The main continuous study variables showed distinct but related patterns (Table 4). Burnout and secondary traumatic stress were strongly positively correlated (r = 0.721). Compassion satisfaction was negatively correlated with secondary traumatic stress (r = −0.350) and GHQ-12 psychological distress (r = −0.487), whereas psychological distress was positively correlated with burnout (r = 0.434) and secondary traumatic stress (r = 0.679). The complete correlation matrix, including sociodemographic and occupational variables, is provided in Supplementary Material S3.

### 3.5. Adjusted Multilevel Associations

In the adjusted random-intercept models, higher psychological distress was consistently associated with less favorable professional quality of life. Higher GHQ-12 scores were associated with lower compassion satisfaction (B = −0.605, 95% CI [−0.674, −0.535], *p* < 0.001), higher burnout (B = 0.407, 95% CI [0.354, 0.461], *p* < 0.001), and higher secondary traumatic stress (B = 0.906, 95% CI [0.843, 0.969], *p* < 0.001) (Table 5). Several secondary sociodemographic or occupational covariates had nominal *p*-values below 0.05, but none of the 27 secondary effects retained q < 0.05 after Benjamini–Hochberg correction (minimum q = 0.163); these associations are therefore treated as exploratory and are not emphasized as independent findings.

Adjusted institutional/residual variances were 3.661/55.771 for compassion satisfaction, 2.839/32.604 for burnout, and 4.730/44.190 for secondary traumatic stress, corresponding to adjusted ICCs of 0.062, 0.080, and 0.097, respectively.

### 3.6. Model Diagnostics and Robustness

Residuals showed departures from normality and some heteroscedasticity, particularly for compassion satisfaction and secondary traumatic stress; however, the GHQ-12 coefficient retained its direction and changed by no more than 3.0% after joint exclusion of observations with |z| > 3 and by no more than 2.1% after separate exclusion of institutions with extreme random intercepts. Thus, the central adjusted GHQ-12 associations were not driven by any single extreme observation or institution (Supplementary Material S2).

## 4. Discussion

### 4.1. Main Findings and Theoretical Interpretation

The study addressed three aims: describing ProQOL dimensions, quantifying institutional clustering, and examining adjusted associations with psychological distress and secondary covariates. Mean scores were 38.1 for compassion satisfaction, 24.1 for burnout, and 14.9 for secondary traumatic stress, underscoring distinct distributions across the three ProQOL dimensions. Unconditional ICC point estimates indicated modest institutional clustering (8.6% for compassion satisfaction, 9.7% for burnout, and 13.9% for secondary traumatic stress); the estimate for secondary traumatic stress was numerically highest, but the variance components were not formally compared.

The correlation pattern confirmed that the ProQOL dimensions are distinct. Burnout and secondary traumatic stress were strongly positively associated, whereas compassion satisfaction was negatively associated with secondary traumatic stress. The positive, albeit weak, association between compassion satisfaction and burnout requires particularly cautious interpretation. These findings indicate that compassion satisfaction is conceptually distinct from professional strain rather than its mirror image. Professional quality of life should therefore be examined through its three components rather than as a single overall score, as proposed by Stamm (2005). This interpretation is consistent with psychometric research supporting a multidimensional ProQOL structure rather than a single global score (Galiana et al., 2017, 2020). Recent evidence in psychologists and in a large Ecuadorian health-care sample likewise shows that compassion satisfaction can coexist with burnout and secondary traumatic stress, supporting a multidimensional interpretation rather than a single favorable-unfavorable continuum (Jaramillo-Cartwright et al., 2025; Kercher et al., 2024).

Psychological distress was the only primary individual-level correlate consistently associated with all three ProQOL dimensions in the adjusted models: higher GHQ-12 scores were associated with lower compassion satisfaction and higher burnout and secondary traumatic stress. This pattern is consistent with the theoretical expectation that broader mental-health strain can coexist with occupation-specific experiences of exhaustion and indirect trauma exposure. The cross-sectional design does not establish temporal direction, and professional strain may also contribute to psychological distress.

Several sociodemographic and occupational covariates showed nominal associations in the adjusted models, but none of the 27 secondary effects remained significant after Benjamini–Hochberg correction (minimum q = 0.163). These findings are therefore treated as exploratory, and detailed causal or mechanistic explanations are not advanced. Sex and marital status have been associated with burnout and compassion fatigue in health and mental-health professionals (Cañadas-De la Fuente et al., 2018; Roy & Naik, 2025), which motivated their inclusion as secondary covariates. Future studies should evaluate potentially relevant occupational characteristics using prespecified hypotheses and direct measures.

### 4.2. Theoretical and Practical Implications

The ProQOL and JD-R frameworks are complementary but address different levels of the occupational experience. ProQOL distinguishes the positive and negative consequences of helping work through compassion satisfaction, burnout, and secondary traumatic stress, whereas JD-R explains how the balance between job demands and resources may contribute to employee strain or sustained engagement. The study contributes multicenter evidence from Ecuador, an underrepresented Latin American context, while the nonprobability design limits population-level generalization. Recent research integrating ProQOL and JD-R in child mental-health services found that specific job demands and resources were differentially associated with burnout, compassion satisfaction, secondary traumatic stress, and turnover intention, reinforcing the value of measuring organizational exposures directly (Aminihajibashi et al., 2024).

Practically, the findings support attention to professional quality of life and psychological distress among institution-based psychologists. Institutions could evaluate confidential access to psychological support and non-diagnostic monitoring of ProQOL at an aggregate level, with clear procedures for interpretation and referral. The present study did not test interventions. Organizational characteristics not directly measured here-including leadership, staffing, supervision, organizational justice, autonomy, and caseload-should therefore be treated as priorities for future research rather than as explanations for the observed institutional differences. Organizational and structural factors have been linked to vicarious trauma and to professional quality of life in mental health settings (Chatham et al., 2024; Sutton et al., 2022), reinforcing the value of measuring leadership, supervision, staffing, and caseload directly. Qualitative evidence from mental health psychology practitioners also highlights peer support, professional boundaries, and targeted training as relevant supports for practitioner wellbeing after collective trauma (Ventouris et al., 2025).

Study strengths include the large sample—1078 psychologists from 92 institutions—coverage of all 24 provinces of Ecuador, and the simultaneous assessment of positive and negative dimensions of professional quality of life. Linear mixed-effects models explicitly accounted for dependence among participants within the same institution, and assumption checks together with sensitivity analyses involving extreme observations and institutions increased the transparency of the analytical strategy.

### 4.3. Limitations and Future Research

Several limitations should guide interpretation. The cross-sectional design precludes causal or temporal conclusions. Nonprobability sampling, voluntary institutional participation, and the absence of institution-specific denominators may have introduced selection bias and prevented calculation of institution-specific response rates. All measures were self-reported, and urban/rural work setting was based on participant self-classification rather than an external standardized geographic criterion. The sample overrepresented urban work settings and should not be generalized uncritically to all Ecuadorian psychologists. Objective workload, trauma exposure, case complexity, leadership, supervision, staffing, and other organizational resources were not measured, and residual confounding and common-method variance remain possible.

The GHQ-12 warrants continued population-specific validation among Ecuadorian psychologists, although its use as a continuous indicator of psychological distress was supported by the present internal evidence and prior Ecuadorian research.

Future studies should use longitudinal and probability-based or more representative sampling designs and directly assess objective and perceived job demands and resources with validated occupation-specific measures. Institution-level research should examine leadership, autonomy, team climate, supervision, staffing, caseload distribution, and trauma exposure and test whether these variables explain or moderate differences in ProQOL dimensions. Longitudinal designs have captured within-person change and protective factors over time and can help clarify temporal direction (Spányik et al., 2023).

## 5. Conclusions

In this multicenter sample of institution-based psychologists in Ecuador, compassion satisfaction, burnout, and secondary traumatic stress showed distinct score distributions. In the adjusted mixed-effects models, greater psychological distress was consistently associated with lower compassion satisfaction and higher burnout and secondary traumatic stress. No sociodemographic or occupational secondary association survived FDR adjustment.

Modest between-institution variability remained, but the study did not measure the organizational characteristics that might account for it. ProQOL may be evaluated as a non-diagnostic monitoring tool alongside appropriate support procedures, while longitudinal research should directly measure institutional demands and resources. Because the study was cross-sectional, nonprobabilistic, and based on self-report, no causal or intervention-effect conclusions can be drawn.

## Figures and Tables

**Table 1:** Participant characteristics.

Characteristic	n (%)	M (SD)
**Sex**		
Men	480 (44.5)	
Women	598 (55.5)	
Age		36 (9.3)
**Marital status**		
Without a partner	497 (46.1)	
With a partner	581 (53.9)	
**Work setting**		
Urban	950 (88.1)	
Rural	128 (11.9)	
**Employment sector**		
Public	661 (61.3)	
Private/mixed	417 (38.7)	
**Contract type**		
Temporary	628 (58.3)	
Permanent	450 (41.7)	
**Employment status**		
Part time	166 (15.4)	
Full time	912 (84.6)	
Monthly income		1930.3 (505.5)
Professional experience		8.1 (7.6)
Daily working hours		9.3 (4.2)
Psychological distress		10.1 (6.8)

Note. Missing data in the analytical sample: n = 0 for all variables shown.

**Table 2:** Continuous ProQOL-IV scores and observed ranges.

Dimension	Mean (SD)	Observed Minimum	Observed Maximum	Analysis
Compassion satisfaction	38.1 (8.8)	0	50	Continuous
Burnout	24.1 (6.6)	0	50	Continuous
Secondary traumatic stress	14.9 (9.6)	0	50	Continuous

Note. N = 1078. All three ProQOL-IV dimensions were analyzed continuously. The observed range was 0–50 for compassion satisfaction, burnout, and secondary traumatic stress; no clinical or diagnostic categories were assigned.

**Table 3:** Variance components and ICCs from the unconditional mixed-effects models.

Dimension	Institutional Variance	Residual Variance	ICC	Institutional Variability (%)
Compassion satisfaction	6.756	71.459	0.086	8.6
Burnout	4.268	39.754	0.097	9.7
Secondary traumatic stress	12.729	79.024	0.139	13.9

Note. N = 1078 participants nested within 92 institutions. Variance components were estimated using restricted maximum likelihood (REML), with a random intercept for institution, a variance-components covariance structure, and the Satterthwaite approximation for degrees of freedom. ICC = institutional variance/(institutional variance + residual variance).

**Table 4:** Bivariate correlations among the principal continuous study variables.

Variable	1	2	3	4
1. Compassion satisfaction	1			
2. Burnout	0.064 *	1		
3. Secondary traumatic stress	−0.350 **	0.721 **	1	
4. Psychological distress (GHQ-12)	−0.487 **	0.434 **	0.679 **	1

Note. N = 1078. Pearson correlations are shown. * *p* < 0.05; ** *p* < 0.01, two-tailed.

**Table 5:** Adjusted fixed-effect estimates from the primary multilevel models.

Predictor	Compassion Satisfaction	Burnout	Secondary Traumatic Stress
Sex: men vs. women	−1.128 [−2.063, −0.193]; *p* = 0.018; q = 0.163	−0.209 [−0.928, 0.510]; *p* = 0.569; q = 0.790	0.805 [−0.034, 1.644]; *p* = 0.060; q = 0.225
Marital status: without vs. with partner	0.382 [−0.628, 1.392]; *p* = 0.459; q = 0.729	0.744 [−0.030, 1.518]; *p* = 0.060; q = 0.225	0.815 [−0.089, 1.719]; *p* = 0.077; q = 0.225
Work setting: urban vs. rural	−1.544 [−3.034, −0.053]; *p* = 0.042; q = 0.225	−0.342 [−1.494, 0.810]; *p* = 0.561; q = 0.790	−1.150 [−2.502, 0.203]; *p* = 0.096; q = 0.235
Contract: temporary vs. permanent	0.075 [−0.941, 1.090]; *p* = 0.886; q = 0.920	0.189 [−0.593, 0.972]; *p* = 0.635; q = 0.790	−0.250 [−1.172, 0.671]; *p* = 0.595; q = 0.790
Employment status: full-time vs. part-time	0.278 [−1.085, 1.640]; *p* = 0.690; q = 0.809	0.937 [−0.118, 1.992]; *p* = 0.082; q = 0.225	0.801 [−0.431, 2.034]; *p* = 0.202; q = 0.455
Sector: public vs. private/mixed	−0.045 [−1.118, 1.029]; *p* = 0.935; q = 0.935	0.352 [−0.487, 1.190]; *p* = 0.411; q = 0.693	0.105 [−0.887, 1.097]; *p* = 0.836; q = 0.903
Monthly income (per USD 100)	0.039 [−0.031, 0.110]; *p* = 0.272; q = 0.490	−0.008 [−0.062, 0.046]; *p* = 0.776; q = 0.873	−0.015 [−0.079, 0.049]; *p* = 0.644; q = 0.790
Daily working hours	0.070 [−0.046, 0.186]; *p* = 0.237; q = 0.465	0.116 [0.026, 0.205]; *p* = 0.011; q = 0.163	0.096 [−0.008, 0.201]; *p* = 0.070; q = 0.225
Professional experience (years)	0.089 [0.019, 0.159]; *p* = 0.013; q = 0.163	0.048 [−0.006, 0.102]; *p* = 0.083; q = 0.225	−0.038 [−0.101, 0.025]; *p* = 0.241; q = 0.465
Psychological distress (GHQ-12)	−0.605 [−0.674, −0.535]; *p* < 0.001	0.407 [0.354, 0.461]; *p* < 0.001	0.906 [0.843, 0.969]; *p* < 0.001

Note. Cells report unstandardized B [95% CI], followed by *p* and, for secondary sociodemographic/occupational effects, Benjamini–Hochberg q. The q values were calculated across 27 secondary effects (nine predictors × three outcomes); none retained q < 0.05. GHQ-12 was the primary psychological predictor and was not included in that BH family. Models included a random intercept for institution and were estimated using REML with the Satterthwaite approximation for degrees of freedom. Monthly income is expressed per USD 100 increase. Model fit (REML): compassion satisfaction −2 restricted log likelihood = 7450.449, AIC = 7454.449, BIC = 7464.415; burnout −2 restricted log likelihood = 6889.515, AIC = 6893.515, BIC = 6903.480; secondary traumatic stress −2 restricted log likelihood = 7223.753, AIC = 7227.753, BIC = 7237.719.

## Data Availability

The anonymized data required to reproduce the primary analyses and the corresponding SPSS syntax are available from the corresponding author upon reasonable request. The dataset is not publicly available because participant consent and the ethics approval require controlled access and protection of confidentiality. Requests will be evaluated by the research team and, where applicable, by the institutional ethics body. Access is restricted to academic, non-commercial purposes and requires a confidentiality agreement. Data will be retained for five years from the date of ethics approval.

## Supplementary Materials

The following supporting information can be downloaded at: https://www.mdpi.com/article/10.3390/bs16091669/s1, Supplementary Material S1: Reproducible IBM SPSS Statistics Syntax for Scoring the Spanish ProQOL-IV; Supplementary Material S2: Diagnostics and Sensitivity Analyses for the Primary Linear Mixed-Effects Models of Compassion Satisfaction, Burnout, and Secondary Traumatic Stress; and Supplementary Material S3: Bivariate Correlations Among the Individual-Level Study Variables. Figure 1 is part of the main manuscript.

## References

[B1-behavsci-16-01669] Aminihajibashi S., Jensen T. K., Skar A.-M. S. (2024). Exploring key job demands and resources in Norwegian child mental health services: A cross-sectional study of associations with and relationship between compassion satisfaction, burnout, secondary traumatic stress and turnover intention. Frontiers in Public Health.

[B2-behavsci-16-01669] Bakker A. B. (2026). The burnout paradox. World Psychiatry.

[B3-behavsci-16-01669] Bakker A. B., Demerouti E. (2007). The Job Demands–Resources model: State of the art. Journal of Managerial Psychology.

[B4-behavsci-16-01669] Ben-Zur H., Michael K. (2007). Burnout, social support, and coping at work among social workers, psychologists, and nurses. Social Work in Health Care.

[B5-behavsci-16-01669] Bride B. E. (2007). Prevalence of secondary traumatic stress among social workers. Social Work.

[B6-behavsci-16-01669] Cañadas-De la Fuente G. A., Ortega E., Ramirez-Baena L., De la Fuente-Solana E. I., Vargas C., Gómez-Urquiza J. L. (2018). Gender, marital status, and children as risk factors for burnout in nurses: A meta-analytic study. International Journal of Environmental Research and Public Health.

[B7-behavsci-16-01669] Chatham A. A., Petruzzi L. J., Patel S., Brode W. M., Cook R., Garza B., Garay R., Mercer T., Valdez C. R. (2024). Structural factors contributing to compassion fatigue, burnout, and secondary traumatic stress among hospital-based healthcare professionals during the COVID-19 pandemic. Qualitative Health Research.

[B8-behavsci-16-01669] Craig C. D., Sprang G. (2010). Compassion satisfaction, compassion fatigue, and burnout in a national sample of trauma treatment therapists. Anxiety, Stress & Coping.

[B9-behavsci-16-01669] Cuartero-Castañer M. E., Hidalgo-Andrade P., Cañas-Lerma A. J. (2021). Professional quality of life, engagement, and self-care in healthcare professionals in Ecuador during the COVID-19 pandemic. Healthcare.

[B10-behavsci-16-01669] Delle Donne V., Massaroni V., Ciccarelli N., Borghetti A., Ciccullo A., Baldin G., Giuliano G., Dusina A., Visconti E., Tamburrini E., Di Giambenedetto S. (2023). Differences in the long-term impact of the COVID-19 pandemic on the mental health and professional quality of life of resident and specialist physicians. La Medicina del Lavoro.

[B11-behavsci-16-01669] Deriglazov D., Halamová J., Kernová L. (2025). Burnout, compassion fatigue, and compassion satisfaction interventions via mobile applications: A systematic review and a meta-analysis. Worldviews on Evidence-Based Nursing.

[B12-behavsci-16-01669] Figley C. R. (1995). Compassion fatigue: Coping with secondary traumatic stress disorder in those who treat the traumatized.

[B13-behavsci-16-01669] Figley C. R. (2002). Compassion fatigue: Psychotherapists’ chronic lack of self-care. Journal of Clinical Psychology.

[B14-behavsci-16-01669] Galiana L., Arena F., Oliver A., Sansó N., Benito E. (2017). Compassion satisfaction, compassion fatigue, and burnout in Spain and Brazil: ProQOL validation and cross-cultural diagnosis. Journal of Pain and Symptom Management.

[B15-behavsci-16-01669] Galiana L., Oliver A., Arena F., De Simone G., Tomás J. M., Vidal-Blanco G., Muñoz-Martínez I., Sansó N. (2020). Development and validation of the short professional quality of life scale based on versions IV and V of the professional quality of life scale. Health and Quality of Life Outcomes.

[B16-behavsci-16-01669] Glowacz F., Schmits E., Kinard A. (2022). The impact of the COVID-19 crisis on the practices and mental health of psychologists in Belgium: Between exhaustion and resilience. International Journal of Environmental Research and Public Health.

[B17-behavsci-16-01669] Goldberg D., Williams P. (1988). A user’s guide to the general health questionnaire.

[B18-behavsci-16-01669] Gómez-Salgado J., Adanaque-Bravo I., Ortega-Moreno M., Allande-Cussó R., Arias-Ulloa C. A., Ruiz-Frutos C. (2021). Psychological distress during the first phase of the COVID-19 pandemic in Ecuador: Cross-sectional study. PLoS ONE.

[B19-behavsci-16-01669] Habtu Y., Kumie A., Selamu M., Kaba M., Harada H., Girma E. (2025). Exploring the link between work-related psychosocial factors and professional quality of life among Ethiopian healthcare workers: Insights from structural equation modelling analyses. PLoS ONE.

[B20-behavsci-16-01669] Hulley S. B., Newman T. B., Cummings S. R., Hulley S. B., Cummings S. R., Browner W. R., Grady D. G., Newman T. B. (2013). Choosing the study subjects: Specification, sampling and recruitment. Designing clinical research.

[B21-behavsci-16-01669] Jaramillo-Cartwright M. J., Mafla-Viscarra A., Izurieta N., Barnett D. J., Hsu E. B., Grunauer M. (2025). Characterizing mental health in an LMIC context: Measuring compassion satisfaction, burnout, and secondary traumatic stress among health care providers in Ecuador during COVID-19 with the ProQOL V5 questionnaire. Disaster Medicine and Public Health Preparedness.

[B22-behavsci-16-01669] Kercher A., Gossage L. (2024). Identifying risk factors for compassion fatigue in psychologists in Aotearoa, New Zealand, during the COVID-19 pandemic. Professional Psychology: Research and Practice.

[B23-behavsci-16-01669] Kercher A., Rahman J., Pedersen M. (2024). The COVID-19 pandemic, psychologists’ professional quality of life and mental health. Frontiers in Psychology.

[B24-behavsci-16-01669] Lobo R., Kumar S. P., TM R. (2024). Professional quality of life among mental health nurses: A systematic review and meta-analysis. International Journal of Mental Health Nursing.

[B25-behavsci-16-01669] Maurya R. K., DeDiego A. C. (2025). Exploring the relationship between compassion, professional identity, and practice-related variables for counselors. Counselling Psychology Quarterly.

[B26-behavsci-16-01669] Morante-Benadero M. E., Moreno-Jiménez B., Rodríguez-Muñoz A. (2006). Professional satisfaction and fatigue subscales—Version IV (ProQOL) *[Spanish translation of the instrument]*.

[B27-behavsci-16-01669] Moreta-Herrera R., López-Calle C., Ramos-Ramírez M., López-Castro J. (2018). Estructura factorial y fiabilidad del Cuestionario de Salud General de Goldberg (GHQ-12) en universitarios ecuatorianos. Revista Argentina de Ciencias del Comportamiento.

[B28-behavsci-16-01669] Ndulue O. I., Chukka A., Naslund J. A. (2024). Burnout and mental distress among community health workers in low- and middle-income countries: A scoping review of studies during the COVID-19 pandemic. Global Health Journal.

[B29-behavsci-16-01669] Niemitz M., Gaber A.-M., Goldbeck L., Wallenwein A., Tutus D., Fegert J. M., Smaczny C., Heuer H.-E., Junge S., Hebestreit H., Schlangen M. (2022). Professional quality of life among health care professionals in cystic fibrosis and child and adolescent mental health. Journal of Workplace Behavioral Health.

[B30-behavsci-16-01669] Pellegrini S., Moore P., Murphy M. (2022). Secondary trauma and related concepts in psychologists: A systematic review. Journal of Aggression, Maltreatment & Trauma.

[B31-behavsci-16-01669] Phu T., Kram S., Tuia E., Manwar A., Pundir P. (2026). Factors associated with compassion fatigue among front line healthcare workers from low- and middle-income countries: A narrative review. Health Sciences Review.

[B32-behavsci-16-01669] Rosales Vaca K. M., Cruz Barrientos O. I., Girón López S., Noriega S., More Árias A., Guariente S. M. M., Zazula R. (2022). Mental health of healthcare workers of Latin American countries: A review of studies published during the first year of the COVID-19 pandemic. Psychiatry Research.

[B33-behavsci-16-01669] Rossi A., Cetrano G., Pertile R., Rabbi L., Donisi V., Grigoletti L., Curtolo C., Tansella M., Thornicroft G., Amaddeo F. (2012). Burnout, compassion fatigue, and compassion satisfaction among staff in community-based mental health services. Psychiatry Research.

[B34-behavsci-16-01669] Roy M., Naik A. R. (2025). A socio-demographic analysis of compassion fatigue among mental health practitioners. Community Mental Health Journal.

[B35-behavsci-16-01669] Ruiz-Frutos C., Arias-Ulloa C. A., Ortega-Moreno M., Romero-Martín M., Escobar-Segovia K. F., Adanaque-Bravo I., Gómez-Salgado J. (2022). Factors associated to psychological distress during the COVID-19 pandemic among healthcare workers in Ecuador. International Journal of Public Health.

[B36-behavsci-16-01669] Santana Ferreira S. M. D., Zaia V. (2025). Burnout, life satisfaction, and work-related quality of life among psychologists. Frontiers in Psychology.

[B37-behavsci-16-01669] Singh J., Karanika-Murray M., Baguley T., Hudson J. (2020). A systematic review of job demands and resources associated with compassion fatigue in mental health professionals. International Journal of Environmental Research and Public Health.

[B38-behavsci-16-01669] Spányik A., Simon D., Rigó A., Griffiths M. D., Demetrovics Z. (2023). Emotional exhaustion and traumatic stress among healthcare workers during the COVID-19 pandemic: Longitudinal changes and protective factors. PLoS ONE.

[B39-behavsci-16-01669] Sprang G., Clark J. J., Whitt-Woosley A. (2007). Compassion fatigue, compassion satisfaction, and burnout: Factors impacting a professional’s quality of life. Journal of Loss and Trauma.

[B40-behavsci-16-01669] Stamm B. H. (2005). The ProQOL manual: The professional quality of life scale: Compassion satisfaction, burnout and compassion fatigue/secondary trauma scales.

[B41-behavsci-16-01669] Stamm B. H. (2010). The concise ProQOL manual: The professional quality of life scale: Compassion satisfaction, burnout, and secondary traumatic stress.

[B42-behavsci-16-01669] Sutton L., Rowe S., Hammerton G., Billings J. (2022). The contribution of organisational factors to vicarious trauma in mental health professionals: A systematic review and narrative synthesis. European Journal of Psychotraumatology.

[B43-behavsci-16-01669] Vaca-Auz J., Revelo-Villarreal S., Anaya-González J. L., Vaca-Orellana C., Castillo R., Altamirano-Zavala G., Vicens-Blanes F., Molina-Mula J. (2024). Psycho-emotional impact of the COVID-19 pandemic on nursing professionals in Ecuador: A cross-sectional study. BMC Nursing.

[B44-behavsci-16-01669] Ventouris A., Wezyk A., Panourgia C. (2025). The impact of collective trauma on mental health psychology practitioners’ wellbeing: Insights gained from COVID-19. SSM–Qualitative Research in Health.

[B45-behavsci-16-01669] von Elm E., Altman D. G., Egger M., Pocock S. J., Gøtzsche P. C., Vandenbroucke J. P. (2007). The Strengthening the Reporting of Observational Studies in Epidemiology (STROBE) statement: Guidelines for reporting observational studies. PLoS Medicine.

[B46-behavsci-16-01669] Yılmaz T., Bekaroğlu E. (2025). Factors related to secondary traumatic stress among psychologists following the 2023 Kahramanmaraş earthquakes. Professional Psychology: Research and Practice.

