# Supplementary material for: Professional Quality of Life Among Institution-Based Psychologists in Ecuador: A Multilevel Cross-Sectional Study of Compassion Satisfaction, Burnout, and Secondary Traumatic Stress
Source: Behav Sci (Basel). 2026 Sep 17;16(9):1669. doi: 10.3390/bs16091669 (PMC13603473; doi:10.3390/bs16091669)
Supplement: Supplementary file 1 [file behavsci-16-01669-s001.zip › Lista STROBE final.pdf]

STROBE Statement - Checklist for a Cross-Sectional Study

Manuscript: Professional Quality of Life among Institution-Based Psychologists in Ecuador: A Multilevel Cross-Sectional Study of Compassion Satisfaction, Burnout, and Secondary Traumatic Stress

Checklist basis: Final clean revised manuscript, 23 rendered pages. Locations below refer to the final revised version.

Section	Item	STROBE recommendation	Location in final manuscript	Assessment
Title and abstract	1(a)	Indicate the study design with a commonly used term in the title or abstract.	p. 1, title and Abstract	Complies. The title identifies a multilevel cross-sectional study and the Abstract describes a multicenter cross-sectional design.
Title and abstract	1(b)	Provide an informative and balanced summary of what was done and the main results.	p. 1, Abstract	Complies. The Abstract reports the evidence gap, design, nonprobability sampling, N = 1,078 in 92 institutions, ProQOL-IV and GHQ-12 measures, mixed-effects models, ICCs, principal adjusted associations, multiplicity result, implications, and limitations.
Introduction	2	Explain the scientific background and rationale for the investigation.	pp. 1-4, Sections 1.1-1.4	Complies. The revised Introduction develops the occupational context, JD-R framework, theoretical and clinical relevance of ProQOL dimensions and psychological distress, magnitude where supported by prior literature, and the Ecuadorian evidence gap.
Introduction	3	State specific objectives and any prespecified hypotheses, when applicable.	p. 4, Section 1.4	Complies. Three objectives and corresponding research questions are stated. No a priori directional hypotheses were specified.
Methods	4	Present key elements of the study design early in the manuscript.	pp. 1 and 4, title/Abstract and Section 2.1	Complies. The design is identified as observational, cross-sectional, multicenter, and institution-based.
Methods	5	Describe setting, locations, and relevant dates, including recruitment and data collection.	pp. 4-5, Sections 2.1-2.3	Complies. Public/private/mixed-sector setting, nationwide territorial coverage strategy, dates (November 2024-March 2025), institutional recruitment, and online administration are described.
Methods	6(a)	Report eligibility criteria and sources/methods of participant selection.	pp. 5-6, Sections 2.2-2.3; pp. 8-10, Results/Figure 1	Complies. Eligibility, nonprobability recruitment, institutional authorization, voluntary participation, and participant flow are reported.
Methods	6(b)	Report matching criteria when a matched design was used.	Not applicable	Not applicable. No participant matching was used.
Methods	7	Clearly define outcomes, predictors, potential confounders, and effect modifiers; give diagnostic criteria if applicable.	pp. 6-8, Sections 2.4 and 2.6	Complies. The three ProQOL-IV dimensions are outcomes; GHQ-12 psychological distress is the primary psychological predictor; nine sociodemographic/occupational variables are prespecified secondary covariates. No clinical diagnoses are assigned.
Methods	8	For each variable, report data source and method of measurement.	pp. 6-7, Section 2.4	Complies. Participant characteristics, ProQOL-IV, and GHQ-12 administration, scoring, and relevant reliability/validity evidence are described.
Methods	9	Describe measures taken to address potential sources of bias.	pp. 5-8, Sections 2.2-2.6; p. 18, Section 4.3	Complies with limitations acknowledged. Standardized procedures, pilot testing, trained research assistants, prespecified cleaning, complete-case analysis, and sensitivity diagnostics are reported; selection and self-report biases are discussed.
Methods	10	Explain how study size was determined.	p. 5, Section 2.2	Complies. The original descriptive sample-size calculation and its assumptions are reported, together with its limitations for model-specific power and representativeness.
Methods	11	Explain how quantitative variables were handled, including groupings when used.	pp. 6-8, Sections 2.4 and 2.6	Complies. ProQOL-IV and GHQ-12 scores are analyzed continuously; monthly income is rescaled per USD 100. Descriptive ranges are not treated as clinical categories.
Statistical methods	12(a)	Describe all statistical methods, including those used to control for confounding.	pp. 7-8, Section 2.6	Complies. Descriptive/correlation methods, random-intercept mixed-effects models, REML, Satterthwaite degrees of freedom, ICCs, confidence intervals, covariate adjustment, and Benjamini-Hochberg correction are described.
Statistical methods	12(b)	Describe subgroup and interaction analyses, if performed.	Not applicable	Not applicable. No subgroup or interaction analyses are reported.
Statistical methods	12(c)	Explain how missing data were addressed.	pp. 7-8, Section 2.6; pp. 8-11, Results/Table 1	Complies. No imputation or score prorating was used; analyses use the same complete-case sample of 1,078.
Statistical methods	12(d)	For cross-sectional studies, explain how the sampling strategy was incorporated into the analysis, when applicable.	pp. 4-5 and 7-8, Sections 2.1-2.2 and 2.6	Complies. The nonprobability sampling frame and limits on representativeness are explicit; within-institution dependence is handled by random intercepts and ICCs.
Statistical methods	12(e)	Describe sensitivity analyses.	p. 8 and p. 15; Supplementary Material S2	Complies. Observation-level and institution-level influence/sensitivity analyses for the final primary models are summarized in the manuscript and reported in Supplementary Material S2.
Results	13(a)	Report numbers of individuals at each stage of the study.	pp. 8-10, Section 3.1 and Figure 1	Complies. The manuscript reports numbers identified/invited, assessed, included, excluded, and analyzed.
Results	13(b)	Give reasons for non-participation or exclusion at each stage.	pp. 8-10, Section 3.1 and Figure 1	Complies. Refusals, eligibility exclusions, duplicates, invalid/inconsistent records, and incomplete questionnaires are reported.
Results	13(c)	Consider use of a flow diagram.	p. 9, Figure 1	Complies. Figure 1 presents the recruitment, eligibility, exclusion, cleaning, and final-sample flow.
Results	14(a)	Describe characteristics of study participants and potential confounders.	pp. 10-11, Table 1	Complies. Sex, age, marital status, work setting, employment sector, contract type, employment status, income, professional experience, working hours, and psychological distress are summarized.
Results	14(b)	Indicate number of participants with missing data for each variable of interest.	pp. 10-11, Table 1 note	Complies. The final analytical dataset is complete-case and Table 1 states n = 0 missing for variables shown.
Results	14(c)	For cohort studies, summarize follow-up time.	Not applicable	Not applicable because the study is cross-sectional.
Results	15	Report outcome events or summary measures.	pp. 11-13, Tables 2-3	Complies. Means, SDs, observed ranges, variance components, and unconditional ICCs are reported for the three ProQOL-IV outcomes.
Main results	16(a)	Give unadjusted and adjusted estimates, precision, and variables adjusted for.	pp. 13-15, Tables 4-5; Section 2.6	Complies. Table 4 provides focused bivariate associations and Table 5 provides adjusted mixed-model coefficients with 95% CIs, p values, and multiplicity-adjusted q values for

				secondary effects.
Main results	16(b)	Report category boundaries when continuous variables are categorized.	Not applicable	Not applicable in the inferential analyses; ProQOL-IV outcomes and GHQ-12 are analyzed continuously.
Main results	16(c)	Translate relative estimates into absolute risks when relevant.	Not applicable	Not applicable. Relative risks and follow-up periods were not estimated.
Other analyses	17	Report other analyses, including sensitivity analyses.	p. 15, Section 3.6; Supplementary Material S2	Complies. Residual diagnostics and observation-/institution-level sensitivity analyses are reported for the final primary models.
Discussion	18	Summarize key results with reference to study objectives.	pp. 16-18, Sections 4.1-5	Complies. The Discussion returns to ProQOL distributions, institutional clustering, psychological-distress associations, and the study objectives.
Discussion	19	Discuss limitations, sources of bias or imprecision, and their direction/magnitude when possible.	p. 18, Section 4.3	Complies. Cross-sectional design, nonprobability sampling, unavailable denominators, possible selection bias, self-report/common-method variance, urban overrepresentation, residual confounding, and unmeasured organizational exposures are discussed.
Discussion	20	Provide a cautious overall interpretation considering objectives, limitations, multiplicity, and prior evidence.	pp. 16-18, Sections 4.1-5	Complies. Interpretation is noncausal, multiplicity is considered, and conclusions are limited to measured constructs and the accessible sample.
Discussion	21	Discuss generalizability or external validity.	pp. 4-5 and p. 18, Sections 2.1-2.2 and 4.3	Complies. Coverage of all provinces is explicitly distinguished from national representativeness, and generalization to all Ecuadorian psychologists is cautioned.
Other information	22	Give the source of funding and the role of the funder.	p. 19, Funding statement	Complies. Universidad Técnica Particular de Loja and grant PROY_INV_PS_2024_4662 are identified; the funder's non-involvement in design, data collection, analysis, interpretation, writing, and publication decision is stated.

Note. STROBE is a reporting-transparency guideline rather than a methodological quality scale. This checklist was updated against the final clean revised manuscript.
