# Supplementary material for: Professional Quality of Life Among Institution-Based Psychologists in Ecuador: A Multilevel Cross-Sectional Study of Compassion Satisfaction, Burnout, and Secondary Traumatic Stress
Source: Behav Sci (Basel). 2026 Sep 17;16(9):1669. doi: 10.3390/bs16091669 (PMC13603473; doi:10.3390/bs16091669)
Supplement: Supplementary file 1 [file behavsci-16-01669-s001.zip › Material suplementario S2 Diagnosticos y sensibilidad.pdf]

# Supplementary Material S2

*Diagnostics and Sensitivity Analyses for the Primary Linear Mixed-Effects Models of Compassion Satisfaction, Burnout, and Secondary Traumatic Stress*

**Study:** Professional Quality of Life among Institution-Based Psychologists in Ecuador: A Multilevel Cross-Sectional Study of Compassion Satisfaction, Burnout, and Secondary Traumatic Stress

## Primary-model specification

The primary random-intercept models included sex, marital status, work setting, contract type, employment status, employment sector, monthly income (per USD 100), daily working hours, professional experience, and GHQ-12 psychological distress as fixed effects, with a random intercept for institution. The analytical sample comprised 1,078 participants nested within 92 institutions. The models reproduced the main-manuscript estimates to published rounding: GHQ-12 B = -0.605 for compassion satisfaction, B = 0.407 for burnout, and B = 0.906 for secondary traumatic stress. The corresponding institutional/residual variances were 3.661/55.771, 2.839/32.604, and 4.730/44.190, respectively. The diagnostic analyses below therefore correspond directly to the final primary model specification.

**Compassion satisfaction.** Conditional residuals had a mean close to zero, SD = 7.297, and a range from -41.221 to 20.947. Residual skewness was -0.960 and excess kurtosis was 1.795; the Shapiro-Wilk test was significant (W = 0.952, $p < .001$), with the Q-Q plot showing the main departure in the lower tail. The residual-versus-fitted plot showed no severe systematic curvature but did show greater dispersion at lower/intermediate fitted values, consistent with moderate heteroscedasticity and the bounded outcome scale. Nine observations had $|z| > 3$ (IDs 148, 305, 462, 528, 535, 556, 585, 713, 728). Across one-at-a-time exclusions, the GHQ-12 coefficient ranged from -0.615 to -0.602; after joint exclusion of all nine observations it was B = -0.618, an absolute relative change of 2.2% from the full revised-primary estimate. Institutional and residual variances changed by -23.2% and -11.9%, respectively. Institutional random intercepts were approximately compatible with normality (W = 0.981, $p = .187$; skewness = -0.467).

**Burnout.** Conditional residuals had a mean close to zero, SD = 5.562, and a range from -21.161 to 25.468. Residual skewness was 0.223 and excess kurtosis was 0.842; normality deviations were comparatively mild but detectable (W = 0.993, $p < .001$). The residual-versus-fitted plot showed no major curvature and only modest changes in dispersion. Ten observations had $|z| > 3$ (IDs 1, 2, 4, 5, 12, 13, 20, 148, 153, 801). Across one-at-a-time exclusions, the GHQ-12 coefficient ranged from 0.402 to 0.416; after joint exclusion it was B = 0.413, an absolute relative change of 1.4%. Institutional and residual variances changed by -12.7% and -10.4%, respectively. The institutional random intercepts showed a lower-tail departure from normality (W = 0.958, $p = .005$; skewness = -0.768), which was evaluated further through institution-level exclusions.

**Secondary traumatic stress.** Conditional residuals had a mean close to zero, SD = 6.460, and a range from -22.384 to 36.796. Residual skewness was 0.899 and excess kurtosis was 2.690; normality was not supported by the Shapiro-Wilk test (W = 0.960, $p < .001$), with the Q-Q plot showing the largest deviation in the upper tail. The residual-versus-fitted plot showed no severe systematic curvature but did show some increase in dispersion across fitted values. Twelve observations had $|z| > 3$ (IDs 1, 2, 3, 4, 5, 6, 7, 8, 9, 10, 12, 20). Across one-at-a-time exclusions, the GHQ-12 coefficient ranged from 0.899 to 0.917; after joint exclusion it was B = 0.933, an absolute relative change of 3.0%. Institutional and residual variances changed by -3.3% and -18.5%, respectively. Institutional random intercepts showed a modest upper-tail departure from normality (W = 0.967, $p = .019$; skewness = 0.610), which was also assessed by institution-level exclusions.

**Figure S1**

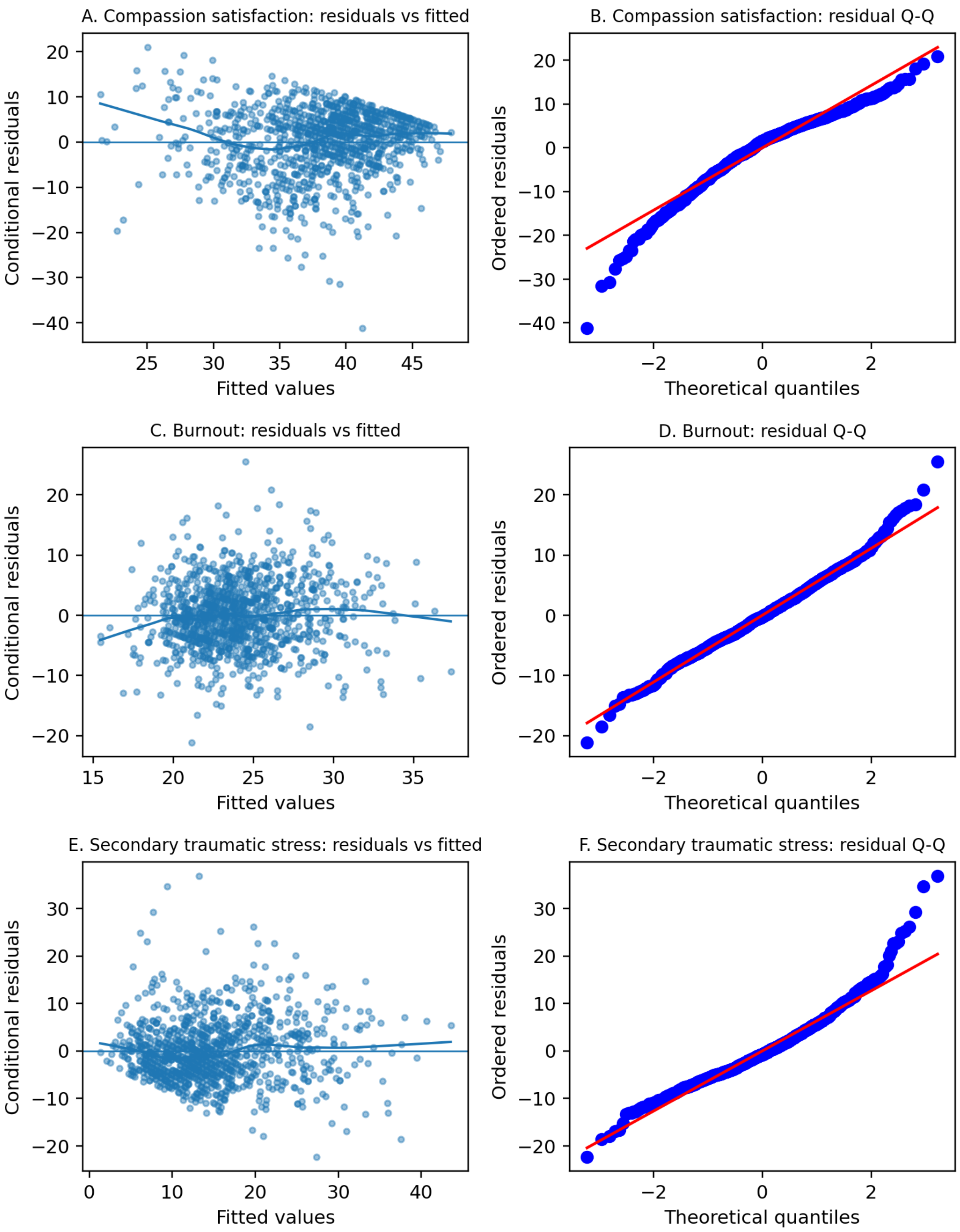


*Diagnostic plots for the primary mixed-effects models. Left panels show conditional residuals versus fitted values with a LOWESS smooth and a horizontal zero reference. Right panels show normal Q-Q plots of the conditional residuals. Panels A-B: compassion satisfaction; C-D: burnout; E-F: secondary traumatic stress.*

**Institution-level sensitivity.** For compassion satisfaction, the two highest and two lowest estimated institutional random intercepts were observed for Institutions 25 (+2.517), 67 (+2.190), 49 (-3.404), 18 (-3.402); excluding each institution separately yielded GHQ-12 coefficients from -0.613 to -0.599 (maximum absolute relative change 1.5%),

while the largest absolute changes in institutional and residual variance were 13.1% and 2.7%. For burnout, the corresponding institutions were 87 (+2.534), 58 (+2.394), 44 (-4.166), 43 (-3.849); GHQ-12 coefficients ranged from 0.406 to 0.416 (maximum change 2.1%). The largest change in the institutional variance component was 24.6%, whereas residual variance changed by at most 1.1%. For secondary traumatic stress, the corresponding institutions were 46 (+4.370), 92 (+4.185), 44 (-3.545), 43 (-3.413); GHQ-12 coefficients ranged from 0.896 to 0.907 (maximum change 1.1%), with maximum absolute institutional and residual variance changes of 13.3% and 0.9%, respectively. Thus, the fixed GHQ-12 associations were not materially altered by exclusion of any institution with an extreme random intercept, although the magnitude of the random variance component was more sensitive in the burnout model.

**Conclusion.** Residuals showed departures from normality and some heteroscedasticity, especially for compassion satisfaction and secondary traumatic stress, consistent with the bounded ProQOL-IV outcome scales. Nevertheless, the GHQ-12 coefficient retained its direction and changed by no more than 3.0% after joint exclusion of observations with $|z| > 3$ and by no more than 2.1% after separate exclusion of institutions with extreme random intercepts. Variance components were more sensitive than the GHQ-12 fixed effect in some analyses. Thus, the central adjusted GHQ-12 associations were not driven by any single extreme observation or institution; all coefficients remain noncausal given the cross-sectional, nonprobability design.
