# Supplementary material for: Professional Quality of Life Among Institution-Based Psychologists in Ecuador: A Multilevel Cross-Sectional Study of Compassion Satisfaction, Burnout, and Secondary Traumatic Stress
Source: Behav Sci (Basel). 2026 Sep 17;16(9):1669. doi: 10.3390/bs16091669 (PMC13603473; doi:10.3390/bs16091669)
Supplement: Supplementary file 1 [file behavsci-16-01669-s001.zip › Material suplementario S3 Correlaciones bivariadas(1).pdf]

Supplementary Material S3

Bivariate Correlations Among the Individual-Level Study Variables

Study: Professional Quality of Life among Institution-Based Psychologists in Ecuador: A Multilevel Cross-Sectional Study of Compassion Satisfaction, Burnout, and Secondary Traumatic Stress

Table S1. Bivariate correlations among the individual-level study variables.

	(1)	(2)	(3)	(4)	(5)	(6)	(7)	(8)	(9)	(10)	(11)	(12)	(13)	(14)
**1. Compassion satisfaction (ProQOL-CS)**	1													
**2. Burnout**	.064*	1												
**3. Secondary traumatic stress (ProQOL-STS)**	-.350**	.721**	1											
**4. Sex (0 = men; 1 = women)**	.062*	.014	-.051	1										
**5. Age**	.113**	-.034	-.138**	-.142**	1									
**6. Marital status (0 = without a partner; 1 = with a partner)**	-.015	-.041	-.042	-.087**	.457**	1								
**7. Work setting (0 = urban; 1 = rural)**	.052	.020	.043	.000	-.093**	-.103**	1							
**8. Employment sector (0 = public; 1 = private/mixed)**	.036	-.084**	-.076*	-.040	.007	-.018	-.091**	1						
**9. Contract type (0 = temporary; 1 = permanent)**	.018	-.004	.000	-.086**	.274**	.206**	-.125**	.019	1					
**10. Employment status (0 = part-time; 1 = full-time)**	.026	-.112**	-.104**	.010	-.056	-.080**	.026	.242**	-.184**	1				
**11. Monthly income**	.036	-.038	-.047	-.052	.098**	.031	.022	-.052	.056	-.029	1			
**12. Professional experience**	.121**	-.045	-.141**	-.107**	.834**	.385**	-.069*	.032	.296**	-.040	.093**	1		
**13. Daily working hours**	-.048	.111**	.125**	-.071*	-.039	-.017	-.017	-.061*	.090**	-.101**	-.054	-.052	1	
**14. Psychological distress (GHQ-12)**	-.487**	.434**	.679**	-.016	-.152**	-.001	.004	-.083**	-.043	-.080**	-.041	-.154**	.118**	1

Note. N = 1,078. Pearson's r is reported for associations between two continuous variables, the point-biserial correlation for associations between a continuous and a dichotomous variable, and the phi coefficient for associations between two dichotomous variables. GHQ-12 = 12-item General Health Questionnaire; ProQOL = Professional Quality of Life Scale. * $p < .05$; ** $p < .01$, two-tailed.
